# Towards sustainable bioplastic production using the photoautotrophic bacterium *Rhodopseudomonas palustris* TIE-1

**DOI:** 10.1007/s10295-019-02165-7

**Published:** 2019-03-29

**Authors:** Tahina Onina Ranaivoarisoa, Rajesh Singh, Karthikeyan Rengasamy, Michael S. Guzman, Arpita Bose

**Affiliations:** Department of Biology, Washington University in St. Louis, One Brookings Drive, St. Louis, MO 63130 USA

**Keywords:** *Rhodopseudomonas palustris* TIE-1, Polyhydroxybutyrate, PHB, Photoferroautotrophy, Photoelectroautotrophy

## Abstract

Bacterial synthesis of polyhydroxybutyrates (PHBs) is a potential approach for producing biodegradable plastics. This study assessed the ability of *Rhodopseudomonas palustris* TIE-1 to produce PHBs under various conditions. We focused on photoautotrophy using a poised electrode (photoelectroautotrophy) or ferrous iron (photoferroautotrophy) as electron donors. Growth conditions were tested with either ammonium chloride or dinitrogen gas as the nitrogen source. Although TIE-1’s capacity to produce PHBs varied fairly under different conditions, photoelectroautotrophy and photoferroautotrophy showed the highest PHB electron yield and the highest specific PHB productivity, respectively. Gene expression analysis showed that there was no differential expression in PHB biosynthesis genes. This suggests that the variations in PHB accumulation might be post-transcriptionally regulated. This is the first study to systematically quantify the amount of PHB produced by a microbe via photoelectroautotrophy and photoferroautotrophy. This work could lead to sustainable bioproduction using abundant resources such as light, electricity, iron, and carbon dioxide.

**Electronic supplementary material:**

The online version of this article (10.1007/s10295-019-02165-7) contains supplementary material, which is available to authorized users.

## Introduction

Polyhydroxybutyrates (PHBs) are the most well-studied members of the polyhydroxyalkanoates (PHAs), which is a family of biodegradable intracellular polyesters produced by several bacteria [38, 45, 67, 79, 85]. Due to its thermoresistance, moldability, and biodegradability, PHB is a promising substitute for conventional petroleum-derived plastics [11]. Because of its biocompatibility, PHB is also used in many medical applications such as drug delivery, reconstructive surgery and bone tissue scaffolding [48]. However, its production is currently underexploited due to high feedstock costs [67]. Heterotrophic microbes can be promising PHB producers as they can use low-cost carbon sources including food wastes such as sugar beet, soy, and palm oil molasses [69]. However, the requirement for a continuous supply of food wastes makes them an infeasible source of carbon. Additional challenges of using food wastes are its sorting, transport, and pre-treatment prior to utilization [42, 54]. Lignocellulose from food [54] and forestry industries [41] or glycerol wastes from biofuel production have also been explored in heterotrophic PHB production [4, 75]. Some studies have used pure substrates such as glucose, acetate, and ethanol [79]. However, due to the requirement of arable land, and direct competition with human food consumption, using these substrates for PHB production is not desirable [17]. These potential limitations of using heterotrophs eventually led to the investigation of autotrophs for PHB production [38].

A handful of studies have demonstrated autotrophs as efficient PHB producers over heterotrophs [30]. A chemoautotrophic hydrogen-oxidizing bacterium *Ideonella* spp. strain O-1 has been shown to produce PHB using industrial exhaust gas containing hydrogen (H_2_), carbon dioxide (CO_2_), and carbon monoxide (CO). The exceptional ability of strain O-1 to grow even at CO concentration of 70% (v/v) without suppression of PHB production made it an attractive candidate to produce PHB using industrial exhaust gas (rich in CO) [76]. However, this route of PHB production may not be ideal because of the high cost of operation, and the risk of explosion associated with the use of H_2_ [14]. A sulfate-reducing bacterium *Desulfococcus multivorans* has also been shown to produce PHB [26], but its slow growth rate can make this process inefficient.

To make PHB bioproduction more efficient, economically viable and sustainable, research on autotrophic PHB production was further extended to photoautotrophs. The ability of photoautotrophs to use solar energy and CO_2_ for biosynthesis makes them unique candidates for efficient PHB synthesis [19, 34, 74]. Under photoautotrophic growth conditions, CO_2_ is fixed using energy harvested from light to generate ATP [9]. The fixed carbon can be used for the biosynthesis of acetyl-CoA, a substrate for PHB synthesis. In addition, photoautotrophs capable of fixing dinitrogen gas (N_2_) using ATP generated by photosynthesis are even more desirable. Moreover, nitrogen limitation has been reported to increase PHB accumulation [34]. Indeed, a recent study reported the suitability of the photoautotrophic organism *Synechocystis* sp. PCC 6714 as a potential host strain for PHB production [34]. However, the PHB amount based on cell mass and volumetric productivity was very low [19].

To produce higher PHB with greater efficiency, research on bacterial PHB synthesis was further expanded to microbial electrosynthesis (MES). This approach is based on the ability of some autotrophs (also called electroautotrophs) to acquire electrons from solid-phase conductive substances (SPCSs) such as electrodes using them as electron donors. This process of using SPCSs as electron donors or acceptors is termed “extracellular electron transfer (EET)” [20, 23, 36, 66]. When microbes use SPCSs as electron donors, the form of EET they use is also called microbial electron uptake (EU). This capability of electroautotrophs has been leveraged to produce value-added multi-carbon products via MES by reducing CO_2_ via either indirect or direct EU. In direct EU, microorganisms attach to the electrode and directly take up electrons from them [3, 6, 25, 52, 53, 65, 70, 78, 87, 88], whereas indirect EU involves transport of electrons by diffusible electron careers such as H_2_, formate or ammonia (either produced electrochemically or added to the reactors) from the electrode to microbes [13, 18, 28, 33, 39, 43, 58, 62, 68, 72, 80]. Indirect EU has been successfully used for PHB bioproduction by the chemoautotroph *Cupriavidus necator* (previously named *Ralstonia eutropha*). Nishio et al. reported that PHB productivity in *C. necator* was enhanced by EET using a biocompatible mediator (2-poly (2-methacryloyloxyethyl phosphorylcholine-co-vinylferrocene) (PMF) in an electrochemical system with an anode that was poised at + 0.6 V vs. the standard hydrogen electrode (SHE). Here, the anode served as an additional electron acceptor for microbial metabolism, resulting in acceleration of glycolysis and hence PHB synthesis [55]. Indirect MES of PHB by *C. necator* using formate as an electron carrier has also been reported recently [12]. To enhance CO_2_ assimilation by *C. necator*, a formate dehydrogenase (FDH)-assisted MES system was constructed, in which FDH catalyzed the reduction of CO_2_ to formate in the cathodic chamber. Formate served as the electron carrier to transfer electrons into *C. necator* generating PHBs [12]. The involvement of mediators in indirect EU lowers the efficiency of product formation. Direct EU in the context of MES is desirable because it omits the extra steps involved in indirect EU [12, 55]. Although a thermodynamic evaluation of bacterial PHB production via MES was proposed nearly a decade ago [64], no pure microbial culture has been known to produce PHBs using MES via direct EU (i.e., without the use of mediators).

Due to the abundance of iron on earth [24], PHB production linked to autotrophy using ferrous iron, Fe(II), as an electron donor could be leveraged for sustainable PHB production. Some Fe(II)-oxidizing chemoautotrophs such as *Gallionella ferruginea* have been reported to accumulate PHBs intracellularly. However, quantitative measurements on PHB production have not been reported for this organism [27, 46, 83]. The use of oxygen as the terminal electron acceptor by these organisms is a challenge because oxygen reacts readily with Fe(II) to oxidize and precipitates it to Fe(III). Therefore, *G. ferruginea* can only oxidize iron under low-oxygen concentrations [46]. Photoautotrophs such as purple bacteria and green sulfur bacteria have been shown to oxidize Fe(II) while fixing CO_2_ using light via a process called photoferroautotrophy [21, 73]. Photoferroautotrophs are more attractive for PHB synthesis because they oxidize Fe(II) in the absence of oxygen. However, thus far PHB accumulation has not been demonstrated during photoferroautotrophic growth.

Here, *Rhodopseudomonas palustris* TIE-1 was chosen as a platform for PHB bioproduction because it demonstrates extraordinary metabolic versatility [6, 32]. TIE-1 can grow chemoheterotrophically in rich medium as well as photoheterotrophically using various organic carbon sources [32]. It can also use several inorganic electron donors such as H_2_ and thiosulfate for photoautotrophic growth [32]. More importantly, TIE-1 is the only genetically tractable bacterium that has the ability to perform photoautotrophy using inorganic electron donors such as Fe(II) (photoferroautotrophy) and a poised electrode (photoelectroautotrophy) [6, 32]. TIE-1 performs direct EU to support photoelectrotrophy [6, 65]. These exceptional abilities make TIE-1 a very promising candidate to study photoautotrophic PHB production under different growth conditions. We assessed PHB production quantitatively on several growth conditions and found that TIE-1 can produce PHBs both photoautotrophically and photoheterotrophically. Among the photoautotrophic growth conditions, the highest PHB electron yield [percentage of the electron (mol) from the substrate that was converted into PHBs] was obtained under photoelectroautotrophy, and the highest specific PHB productivity was obtained under photoferroautotrophy. These novel routes of PHB synthesis by TIE-1 can potentially serve as a stepping stone for future bioengineering efforts towards sustainable PHB bioproduction.

## Materials and methods

### Bacterial strain, media, and growth conditions

*Rhodopseudomonas palustris* TIE-1 was originally isolated by Jiao et al. and has been used throughout this study [32]. For aerobic chemoheterotrophic growth, TIE-1 was routinely grown in 0.3% yeast extract and 0.3% peptone (YP) medium, with 10 mM MOPS [3-*N* (morpholino) propanesulfonic acid] at pH 7 in the dark at 30 °C with shaking at 250 rpm. For growth on solid medium, YP medium was solidified with 1.5% agar supplemented with 10 mM MOPS and 10 mM sodium succinate. For anaerobic photoautotrophic growth, TIE-1 was grown in anaerobic bicarbonate-buffered freshwater (FW) medium [21] supplemented with ammonium chloride (NH_4_Cl) (5.61 mM) or mixed N_2_/CO_2_ (80%/20%) gas at a pressure of 34.5 kPa as the sources of nitrogen. For anaerobic photoheterotrophic growth, 10 mL of FW medium was supplemented with anoxic 1 M stocks of sodium succinate, sodium butyrate and sodium 3-hydroxybutyrate to a final concentration of 1 mM in Balch tubes. However, to have higher biomass required for PHB, RNA and protein extraction, substrate concentrations were increased to 10 mM. A pre-grown TIE-1 culture with optical density (OD_660_) of 1 was inoculated with a final OD_660_ of 0.01 (100 × dilution) followed by incubation at 30°C in an environmental chamber fitted with infrared LED (880 nm). Time-course cell growth was monitored using Spectronic 200 (Thermo Fisher Scientific, USA). For photoautotrophic growth with H_2_ and Fe(II), TIE-1 was adapted to photoautotrophic growth using H_2_ as the sole electron donor as described previously [7]. For growth with Fe(II), 50 mL of FW medium was prepared under the flow of 34.5 kPa N_2_/CO_2_ (80%/20%) and dispensed into pre-sterilized serum bottles purged with 34.5 kPa N_2_/CO_2_ (80%/20%). The bottles were then sealed using sterile butyl rubber stoppers with aluminum crimp followed by the addition of anoxic sterile stocks of FeCl_2_ and nitrilotriacetic acid (NTA) to a final concentration of 5 mM and 10 mM, respectively. All sample manipulations were performed inside an anaerobic chamber with 5% H_2_/75% N_2_/20% CO_2_ (Coy laboratory, Grass Lake) [7]. The bacterial generation time was determined as described previously [77]. Lag time (lag) was determined as a period that precedes the exponential phase [47].

### Cell enumeration

Samples were fixed with paraformaldehyde (20% v/v), transferred into Amicon centrifuge filters (Amicon Ultracel 100 k, regenerated cellulose membrane, Millipore, Carrigtwohill, CO, Ireland) and centrifuged for 10 min at 1000×*g*. The pellets were resuspended and washed twice in PBS (phosphate-buffered saline). The cells were recovered by centrifugation at 3000×*g* for 15 min. After the addition of PicoGreen^®^ (Quant-iT PicoGreen^®^ dsDNA, Life Technologies, Grand Island, NY, USA), the cells were counted in 96-well plates along with 50 μL of Sphero™ AccuCount blank beads (Spherotech, Lake Forest, IL, USA). Cell density was estimated with an LSRII flow cytometer (BD, Sparks, MD, USA) using a 488-nm laser. A calibration curve relating the ratio of cell events to bead events and the cell density was constructed using a serial dilution of a cell sample. Density was then determined by microscopy (Helber Bacteria Cell counting chamber with Thoma ruling, Hawksley, Lancing, Sussex, UK). The OD_660_ of TIE-1 cells vs. cell numbers were plotted to obtain a standard curve.

### Bioelectrochemical setup and growth conditions

All photoelectroautotrophic experiments were performed using a three-electrode configured seal-type bioelectrochemical cell (BEC, C001 Seal Electrolytic cell, Xi’an Yima Opto-electrical Technology Com., Ltd, China). The three electrodes were configured as the working electrode (graphite rod, 3.2 cm^2^), reference electrode (Ag/AgCl in 3 M KCl) and counter electrode (Pt foil, 5 cm^2^). 70 mL of FW medium was dispensed into sterile BECs and made completely anaerobic by N_2_/CO_2_ (80%/20%) bubbling for 60 min with the final pressure maintained at ~ 50 kPa. 10 mL of TIE-1 cells (OD_660_ ~ 2.4) pre-grown in FW with H_2_ was then inoculated with a starting OD_660_ ~ 0.3 as described previously [65]. The OD_660_ of the inoculated BECs was monitored with a BugLab Handheld OD Scanner (Applikon Biotechnology, Inc., Foster City, CA). To evaluate the influence of NH_4_Cl and N_2_ gas as the nitrogen sources on PHB biosynthesis via photoelectroautotrophy, the BECs were operated simultaneously (*c* = 3 biological replicates) with NH_4_Cl and N_2_ gas as nitrogen sources with negative controls: open-circuit (OC) control (no current) and abiotic controls. The graphite electrode was constantly poised at a potential of + 100 mV vs. standard hydrogen electrode (SHE) for 130 h using a multichannel potentiostat (Interface 1000E, Gamry Multichannel Potentiostat, USA). All photoelectroautotrophic experiments were performed at 26 °C under continuous infrared light (880 nm) unless noted otherwise. At the end of the bioelectrochemical experiment, samples were immediately collected from the BEC reactors for RNA extraction and PHB production analysis as mentioned above.

### Analytical procedures

*PHB measurement* From all the growth conditions tested, 10 mL of bacterial samples at an OD_660_ 0.7 (unless stated otherwise) was pelleted at 8000×*g* for 10 min and stored at − 80 °C until PHB extraction and analysis were performed. 1 mL of water (LC–MS grade) and 600 µL of methanol (HPLC grade) were added to arrest metabolic activity of TIE-1. 10 mg/mL of poly[(*R*)-3-hydroxybutyric acid] (Sigma-Aldrich, USA) was used as a PHB standard. Extraction of PHB was followed by its conversion to crotonic acid. The concentration of crotonic acid was measured using an Agilent Technologies 6420 Triple Quad LC/MS as follows: using Hypercarb column, particle 5 µm, 100 × 2.1 mm (Thermo Fisher Scientific, USA) as stationary phase; water with 0.1% (v/v) formic acid as phase A; acetonitrile and 1% (v/v) formic acid as phase B. The injection volume was 5 µL; the flow rate was set at 500 µL min^−1^; the column temperature was set at 15 °C and the gas temperature was 300 °C [31]. PHB was detected as crotonic acid with mass to charge ratio (*m*/*z*) = 87 which was normalized to bacterial cell number. Details on PHB extraction, PHB carbon yield, and PHB electron yield calculations are described in supplemental methods.

*H*_*2*_*and CO*_*2*_*measurement* Time-course H_2_ and CO_2_ from photoautotrophic conditions were analyzed using gas chromatography (Shimadzu BID 2010-plus, equipped with Rt^®^-Silica BOND PLOT Column: 30 m × 0.32 mm; Restek, USA) with helium as a carrier gas. At each time point, 10 µL of gas was sampled from the headspace of the serum bottles using a Hamilton™ gas-tight syringe and injected into the column. To quantify dissolved CO_2_, 1 mL of filtered (using 0.22 µm PES membrane filter) aqueous samples from each reactor was collected and injected into helium-evacuated 12-mL septum-capped glass vials (Exetainer, Labco, Houston, TX, USA) containing 1 mL of 85% phosphoric acid. The concentration of the dissolved CO_2_ was then measured by injecting 10 µL of evolved CO_2_ in the headspace into the column. The total CO_2_ in the reactors was calculated as described previously [50].

*Organic acid measurement* Time-course consumption of organic acids such as sodium succinate, sodium butyrate, and sodium 3-hydroxybutyrate under photoheterotrophic conditions were quantified using an Ion Chromatography Metrohm 881 Compact Pro using a Metrosep organic acid column (250 mm length). 0.5 mM H_2_SO_4_ with 15% acetone was used as eluent at a flow rate of 0.4 mL min^−1^ with suppression (10 mM LiCl regenerant).

*Fe(II) measurement* Time-course Fe(II) concentration was measured using the Ferrozine Assay as described previously [7].

*Total protein measurement* Total protein during photoferroautotrophy was measured using trichloroacetic acid (TCA) precipitation as follows: total protein from 2 mL culture (at time point zero and at 192 h for the growth with NH_4_Cl and 360 h with N_2_ gas) in microcentrifuge tube was precipitated using 500 µL 100% TCA. This mixture was incubated for 10 min at 4 °C and centrifuged at 18,000×*g* for 30 min at 4 °C. The pellet was washed with 200 µL cold acetone and centrifuged at 18,000×*g* for 10 min at 4 °C. The pellet was then dried at 95 °C for 10 min to remove any residual acetone and resuspended in 50 µL HCl buffered with 100 mM Tris–Cl, pH 8.0. The BCA (bicinchoninic acid) Protein Assay Kit was employed using the microtiter plate method for protein estimation as specified by the manufacturer’s protocol following TCA precipitation (Thermo Scientific, Waltham, MA). Total protein was measured at an absorbance of 562 nm using the Biotek Synergy HTXmicrotiter plate reader [7]. For a total protein to OD_660_ conversion, total protein of known OD_660_ values of TIE-1 cells was quantified. A standard curve was obtained by plotting OD_660_ vs. total protein measured.

### RNA extraction and sequencing

5 mL of bacterial culture were collected at an OD_660_ ~ 0.7. The RNA was stabilized using 5 mL RNAlater (Qiagen, USA) (buffer that stabilizes and protects RNA from degradation) and incubated at room temperature for 10 min. Bacterial cells were centrifuged at 5000 × *g* for 10 min and pellets were stored at − 80 °C until RNA extraction was performed. RNA extraction was performed using the RNeasy Mini Kit (Qiagen, USA) following the manufacturer’s protocol. DNA removal was performed using the Turbo DNA-free Treatment and Removal Kit (Ambion, USA). DNA contamination was tested using PCR using the primers listed in Table S1 as previously described [6, 7]. Illumina unpaired 150-bp libraries were prepared and sequenced at the Genome Technology Access Center, Washington University on an Illumina MiSeq platform (Illumina Inc., San Diego, CA, USA). Trimmomatic (version 0.36) was used to remove Illumina sequencing adapters, quality trim deteriorating bases (threshold = 20), and length filter (min = 60 bp) [5]. Preprocessed RNA-seq reads were mapped to the published *R. palustris* TIE-1 genome using TopHat2 (version 2.1.1) (https://genomebiology.biomedcentral.com/articles/10.1186/gb-2013-14-4r36) and the gff3 annotation file as a guide for sequence alignment. Bowtie 2 (version 2.3.3.1). (https://www.ncbi.nlm.nih.gov/pmc/articles/PMC3322381/) was used to index the reference genome FASTA file. The number of reads mapping to each feature was counted by HTSeq (version 0.9.1). Differentially expressed genes were predicted in DESEQ 2 (version 1.16.1) using the HTSeq (https://www.ncbi.nlm.nih.gov/pubmed/25260700) read counts and an adjusted *p* value cutoff of 0.05. Heat maps were drawn in R using ggplot2 [44, 82].

### Reverse transcription quantitative PCR analysis (RT-qPCR)

cDNA template was synthesized using the purified RNA samples using the iScript cDNA Synthesis Kit (Biorad, USA). Primers listed in (Table S2) were designed using primer3 software (http://bioinfo.ut.ee/primer3/). RT-qPCR was performed using Biorad CFX connect Real-Time System Model # Optics ModuleA using the following thermal cycling conditions: 1 cycle at 95 °C for 3 min and 30 cycles of 95 °C for 3 s, 60 °C for 3 min, and 65 °C for 5 s according to the manufacturer’s protocol. Fold change comparison and standard deviation calculations were performed as described previously [2].

### Identification of PHB cycle genes of TIE-1

The available TIE-1 genome in the JGI Genome Portal (https://genome.jgi.doe.gov/) was used to search for homolog genes involved in the PHB cycle using Blast search.

### Scanning transmission electron microscopy–electron energy loss spectroscopy (STEM–EELS)

TIE-1 grown under sodium butyrate, Fe(II)–NTA and poised graphite electrode was used as representative samples for STEM-EELS. Briefly, 5 mL planktonic cell suspensions were centrifuged at 6000×*g* for 5 min. followed primary fixation by resuspending the cells pellets in 2% formaldehyde and 2.5% glutaraldehyde in 0.05 M sodium cacodylate buffer (pH 7.2) for ~ 45 min at room temperature. After agar encapsulation followed by primary fixation for ~ 20 min, agar cubes were subjected to secondary fixation for ~ 5 h followed by acetone dehydration and resin infiltration. Ultrathin sections (~ 50–60 nm) were obtained using Reichert Ultracut UCT ultramicrotome (Donald Danforth Plant Science Center, Saint Louis, MO), then mounted directly on amorphous-carbon film-coated TEM Cu-grids. Intracellularly localized PHB granules were characterized using a JEOL JEM-2100F field emission scanning transmission electron microscopy (FE-STEM) with an accelerating voltage of 200 keV (Institute of Material Science and Engineering, WUSTL); the microscope is attached with a Gatan 805 BF/DF detector, Gatan 806 HAADF detector and Gatan 863 Tridiem imaging filter (GIF) system. Images were obtained in STEM mode using HAADF detector and BF detector. EELS spectral images were acquired through working HAADF and GIF jointly. Carbon-K edge and nitrogen-K edge elemental maps were retrieved from STEM–EELS spectral images.

### Statistical analysis

The *P* values were determined by one-way ANOVA followed by a pairwise test with Bonferroni adjustment. For the pairwise test with Bonferroni adjustment, the cutoff *P* value is equal to 0.025

### Nucleotide sequence accession numbers

All RNAseq datasets have been deposited in NCBI under BioProject accession number PRJNA417278.

## Results and discussion

### *Rhodopseudomonas palustris* TIE-1 possesses putative PHB cycle genes

**Fig. 1:**
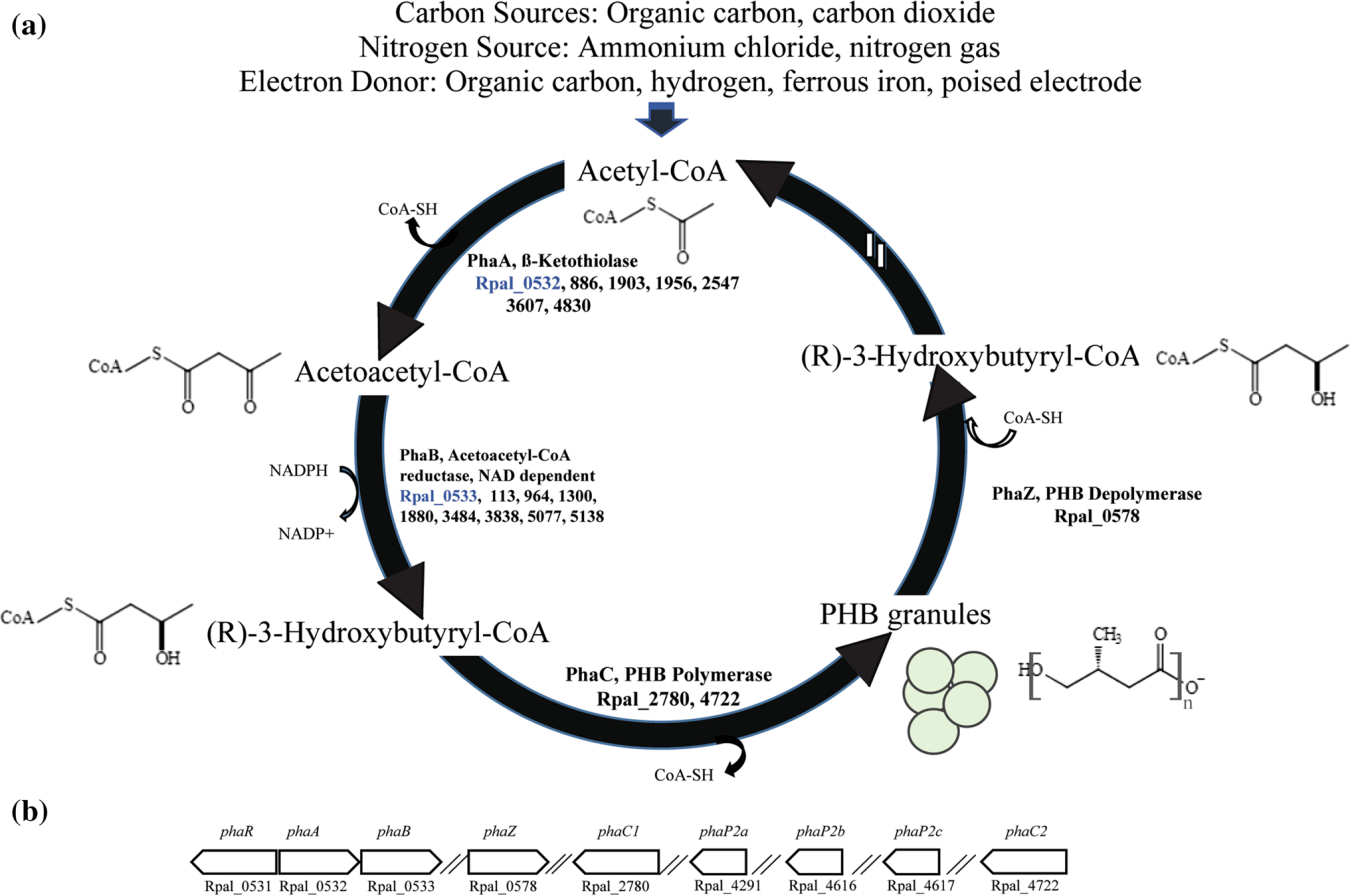
The PHB cycle and its putative genes in TIE-1. **a** Putative PHB cycle of TIE-1. Acetyl-CoA is produced using multiple carbon sources such as organic carbon or carbon dioxide (CO_2_) followed by the condensation of two acetyl-CoAs into acetoacetyl-CoA. Acetoacetyl-CoA gets reduced to (*R*)-3-hydroxybutyryl-CoA by the enzyme acetoacetyl-CoA reductase, PhaB. (*R*)-3-Hydroxybutyryl-CoA is eventually polymerized into PHB granules by PhaC_1_ and/or PhaC_2_. During carbon storage mobilization, PHB is degraded back to (R)-3-hydroxybutyryl-CoA, and then to acetyl-CoA by multiple enzymatic reactions (shown by the double white lines). **b** Organization of the genes involved in the putative PHB cycle in TIE-1. Adapted from [81]

PHB production has been previously reported by several *R. palustris* strains [15, 51, 84]. However, the PHB cycle genes have not been explored thus far. This lack of information critically limits the potential prospects for future bioengineering efforts for PHB bioproduction. Availability of the TIE-1 genome allowed us to identify the genes that are homologous to the PHB cycle genes of *C. necator* [59, 60, 81], an H_2_-oxidizing betaproteobacterium that is known to produce and sequester PHAs intracellularly [8]. Reconstruction of the PHB cycle in *E. coli* using PHB genes from *C. necator* has been previously used to elucidate the biochemical pathway of PHB production and its subsequent metabolism [81]. Briefly, the pathway starts with the condensation of two acetyl-CoAs into acetoacetyl-CoA, a reaction driven by the β-ketothiolase enzyme, PhaA [81] (Fig. 1a). Acetoacetyl-CoA gets reduced to (*R*)-3-hydroxybutyryl-CoA by the enzyme acetoacetyl-CoA reductase, PhaB. Eventually, PHB polymerization is achieved by the PHB polymerase, PhaC (Fig. 1a). Interestingly, PHB can also serve as an important source of carbon and energy during environmental stress, where PHB molecules are catabolized by the PHB depolymerase, PhaZ (Fig. 1a) [81]. In *Bradyrhizobium diazoefficiens*, a nitrogen-fixing symbiont closely related to TIE-1, PhaR represses the expression of *phaC*_*1*_ and *phaC*_*2*_. In addition, PhaR regulates PhaP, the phasin protein that binds to and controls the number and size of the PHB granules. PhaR also binds to PHB granules and dissociates from it as the granule size grows [63]. TIE-1 encodes one *phaR* gene (Rpal_0531); one *phaZ* gene (Rpal_0578); multiple copies of *phaA* and *phaB*; two copies of *phaC* (Rpal_2780 and Rpal_4722); and three genes for *phaP*_*2*_ (Rpal_4291, 4616 and 4617) (Fig. 1a, b). A *phaB* gene (Rpal_0533) is located next to the *phaA* gene (Rpal_0532) (Fig. 1b) forming a putative operon (Fig. 1b). The gene for *phaR* (Rpal_0531) is positioned next to the *phaA* (Rpal_0532) gene in this operon but expressed from the opposite strand. This shows that TIE-1 possesses all the necessary genes for both PHB biosynthesis, polymerization and depolymerization.

### TIE-1 produces PHB under photoautotrophic conditions using different electron donors

#### Photoautotrophic PHB production using H_2_ as an electron donor

**Fig. 2:**
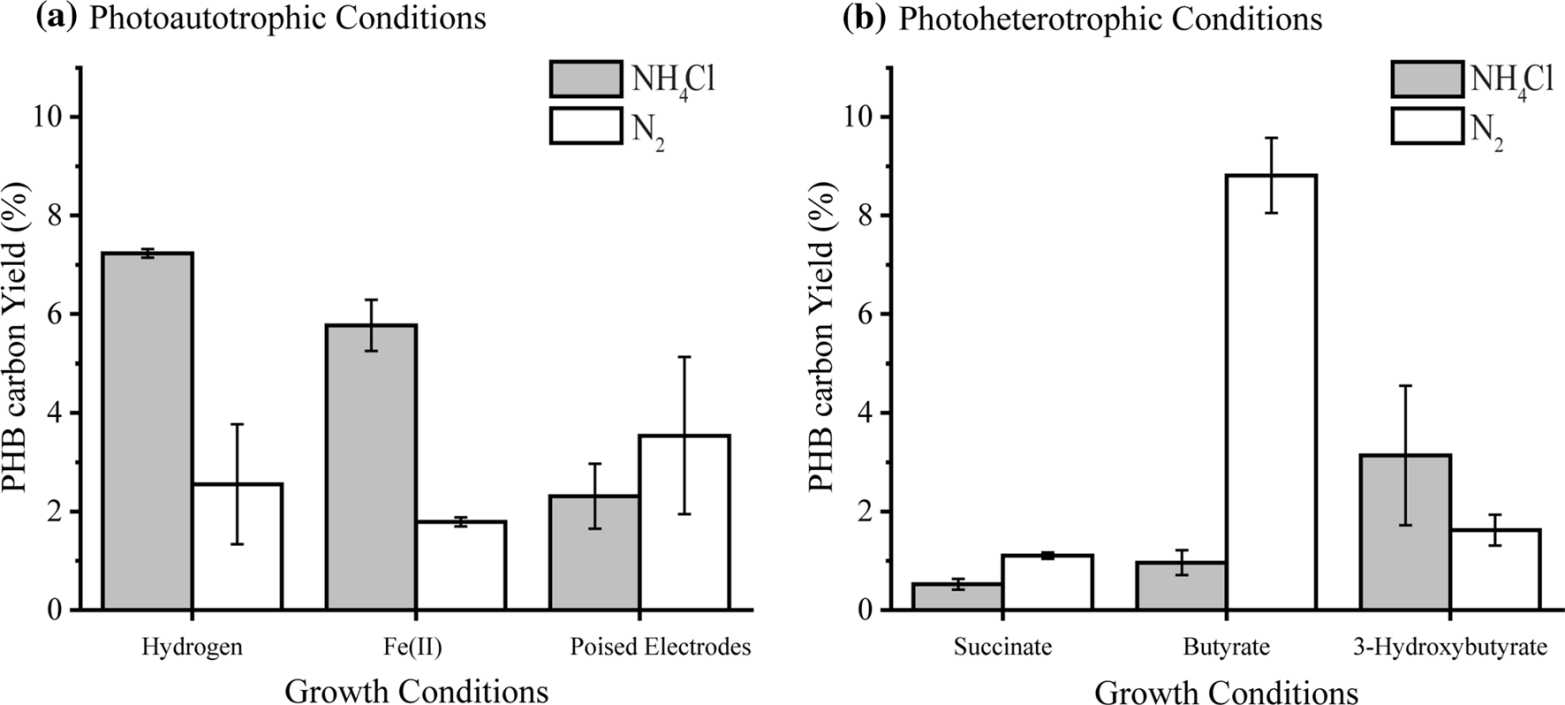
PHB carbon yield by TIE-1 grown in freshwater (FW) medium with ammonium chloride (NH_4_Cl) or under N_2_ fixing conditions (N_2_ gas). **a** Photoautotrophic conditions with H_2_, Fe(II) (photoferroautotrophy) or poised electrodes (photoelectroautotrophy) as electron donors; **b** photoheterotrophic conditions with succinate, butyrate or 3-hydroxybutyrate as electron donors. Error bars are from the standard deviations calculated using 2–3 biological replicates as specified in Table 2. The *P* values were determined by one-way ANOVA followed by a pairwise test with Bonferroni adjustment; *ns* not significant. For a pairwise test, the cutoff *P* value is 0.025. *P* values are indicated in Tables 2, S5

TIE-1 was grown with H_2_ as the sole electron donor with N_2_ and NH_4_Cl as fixed nitrogen sources. We call the N_2_ fixing conditions as the electron donor–N_2_ system and the NH_4_Cl conditions as the electron donor–NH_4_Cl system throughout. TIE-1 showed a higher maximum OD_660_ of 1.16 (*P* ≤ 0.001, Table 1) with NH_4_Cl (H_2_–NH_4_Cl system) compared to OD_660_ of 0.54 with N_2_ (H_2_–N_2_ system). This growth defect observed in H_2_–N_2_ was also reflected in the longer generation time of 41 h in the H_2_–N_2_ system compared to 34 h in the H_2_–NH_4_Cl system (*P *=0.005, Table 1). Slow growth under N_2_ fixing conditions was previously observed in *R. palustris* strain 42 OL [15] and is likely due to the high-energy requirement of this process. TIE-1 showed a lower PHB carbon yield [percentage of carbon (mol) from the substrate that was converted into PHBs] of 2.55% in the H_2_–N_2_ system compared to the ~ 3 times higher yield of 7.23% in the H_2_–NH_4_Cl system. In contrast, no significant difference was observed in PHB electron yield [percentage of the electron (mol) from the substrate that was converted into PHBs] between the H_2_–N_2_ system and the H_2_–NH_4_Cl system (Fig. 2a, Table 2). Interestingly, the specific PHB productivity almost doubled in the H_2_–N_2_ system compared to the H_2_–NH_4_Cl system (from 1.30 × 10^−14^ to 3.08 × 10^−14^ mg/L/Cell/h) (Table 2). The higher specific productivity under N_2_ fixing conditions and the growth defect (Table 1) indicate a direct impact of stress caused by the high-energy-consuming N_2_ fixation process. This stress might have induced TIE-1 to accumulate intracellular PHBs as a carbon and energy reserve. Similar observations have been reported for other purple non-sulfur bacteria [29] grown under nitrogen-limited conditions, including *R. palustris* strains grown in nitrogen-deprived conditions [51]. Increased accumulation of PHB to over 30% of the dry cell weight was observed previously when *R. palustris* CGA009 cells were N_2_ starved [51]. Because H_2_ is known to be produced during N_2_ deprivation by CGA009 [51], this H_2_ could serve as an additional electron donor for photoautotrophy, accounting for additional PHB accumulation. Further studies will be required to test whether this is happening in CGA009 and TIE-1. Although the growth with NH_4_Cl had N_2_ gas in the headspace under most conditions in our experiment, the presence of NH_4_Cl has been known to inhibit nitrogenase gene expression, preventing N_2_ fixation from occurring in the presence of NH_4_Cl [40].

**Table 1:** *R. palustris* TIE-1 growth in different conditions

Growth conditions	Lag time (h)	*P*	Generation time(h)	*P*	Max OD_660_	*P*	Time to achieve max OD_660_ (h)	*P*
YP	16 (0.0)		11.18 (1.1)		0.43 (0.01)		58 (0.0)	
Succinate (NH_4_Cl)	34.6 (6.1)	5.4 × 10^−5^	7.7 (0.4)	0.218	0.27 (0.01)	0.669	79 (2.3)	2.11 × 10^−6^
Succinate (N_2_)	117.3 (5.0)		8.7 (1.1)		0.27 (0.00)		138 (0)	
Butyrate (NH_4_Cl)	21.3 (2.3)	0.11 × 10^−5^	5.3 (0.2)	0.01	0.42 (0.03)	0.0023	62 (0.0)	0.60 × 10^−6^
Butyrate (N_2_)	110.6 (2.3)		7.1 (0.5)		0.69 (0.00)		137 (2)	
3-Hydroxybutyrate (NH_4_Cl)	10.6 (2.3)	3.23 × 10^−5^	8.4 (1.1)	1	0.34 (0.02)	0.946	70 ± 0	3.19 × 10^−6^
3-Hydroxybutyrate (N_2_)	88.6 (6.11)		14.6 (5.8)		0.34 (0.030		119 (2)	
H_2_ (NH_4_Cl)	25.3 (2.3)	1.30 × 10^−5^	34 (7.0)	0.005	1.16 (0.01)	0.000	428 (0)	0.001
H_2_ (N_2_)	60.0 (0)		41 (5.0)		0.54 (0.04)		308 (28)	
Iron (II) (NH_4_Cl)	NM (1)	NM (1)	27.4 (0.6)	7.48 × 10^−5^	0.25 (0.02)	0.006	192 (0.00)	0.000
Iron (II) (N_2_)	NM (1)		49.1 (15.9)		0.14 (0.02)		360 (0.00)	
Photoelectroautotrophy (NH_4_Cl)	NM (2)	NM (2)	76 (10.0)	0.086	0.73 (0.06)	0.021	96 (0.00)^a^	0
Photoelectroautotrophy (N_2_)	NM (2)		82 (8.0)		0.61 (0.01)		96 (0.00)^a^	
Photoelectroautotrophy open circuit (NH_4_Cl)	NM (3)	NM (3)	NM (3)	NM	0.25 (0.014)	0.066	96 (0.00)^a^	0
Photoelectroautotrophy open circuit (N_2_)	NM (3)		NM (3)		0.22 (0.014)		96 (0.00)^a^	

NM (1) = not measurable, bacterial measurements were performed at time zero and time final by total protein conversion to OD; hence lag time could not be measured. NM (2) = not measurable; bacteria were preadapted to hydrogen growth and the inoculum was higher, which led to cells immediately entering exponential phase without lag time. NM (3) = not measurable; bacteria were incubated in the absence of electron donor. Therefore, no growth was observed

*NH*_*4*_*Cl* with ammonium chloride, *N*_*2*_ nitrogen-fixing condition

*P* values are between growth with NH_4_Cl and N_2_ fixing conditions

^a^The experiment was terminated after 96 h, () = standard deviation values from *n* = 3

**Table 2:** PHB production by *R. palustris* TIE-1 under different growth conditions

Growth conditions	PHB (mg/L)	*P*	PHB (mg/L/cell) (× 10^−12^)	*P*	PHB carbon yield (%)	*P*	PHB electron yield (%)	*P*	Specific PHB productivity (mg/L/Cell/h) (× 10^−14^)	*P*
YP	4.37 (0.47)		8.85 (0.94)		NM (1)		NM (1)		24.60 (2.67)	
Succinate (NH_4_Cl)	1.57 (0.20)	0.039	1.45 (0.07)	0.005	0.52 (0.10)	0.001	0.13 (0.00)	0.014	2.55 (0.13)	0.14
Succinate (N_2_)	2.20 (0.35)		2.31 (0.24)		1.10 (0.06)		0.18 (0.01)		3.64 (1.05)	
Butyrate (NH_4_Cl)	3.38 (0.31)^a^	0.001	2.85 (0.31)^a^	0.005	0.96 (0.25)^a^	0.000	0.14 (0.02)^a^	0.038	6.35 (0.69)^a^	0.009
Butyrate (N_2_)	16.57 (1.68)		17.1 (2.68)		8.81 (0.76)		0.77 (0.23)		21.20 (3.31)	
3-Hydroxybutyrate (NH_4_Cl)	2.65 (0.06)^a^	0.057	2.66 (0.25)^a^	0.001	3.13 (1.41)^a^	0.147	NM (2)	NM (2)	6.21 (0.59)^a^	0.525
3-Hydroxybutyrate (N_2_)	5.93 (1.46)		5.29 (0.21)		1.62 (0.43)		NM (2)		6.68 (0.79)	
H_2_ (NH_4_Cl)	5.28 (1.26)^a^	0.686	4.98 (0.65)^a^	0.13	7.23 (0.08)^a^	0.032	0.26 (0.10)^a^	0.681	1.30 (0.05)^a^	0.015
H_2_ (N_2_)	5.26 (1.28)^a^		6.65 (0.68)^a^		2.55 (1.21)^a^		0.22 (0.02)^a^		3.08 (0.31)^a^	
Iron (II) (NH_4_Cl)	4.80 (0.77)^a^	0.029	14.1 (0.00)^a^	0.001	5.77 (0.52)^a^	0.008	0.093 (0.00)^a^	0.048	8.4 (0.05)^a^	0.000
Iron (II) (N_2_)	1.46 (0.26)^a^		7.58 (0.30)^a^		1.79 (0.09)^a^		0.1 (0.00)^a^		5.59 (0.02)^a^	
Photoelectroautotrophy (NH_4_Cl)	4.48 (0.11)^a^	0.17	5.47 (0.13)^a^	0.741	2.30 (0.66)^a^	0.419	4.39 (0.11)^a^	0.064	5.68 (0.14)^a^	0.73
Photoelectroautotrophy (N_2_)	5.49 (0.68)^a^		5.82 (1.29)^a^		3.53 (1.5)^a^		7.34 (1.1)^a^		6.06 (1.34)^§^	
Photoelectroautotrophy open circuit (NH_4_Cl)	ND	ND	ND	ND	ND	ND	ND		ND	ND
Photoelectroautotrophy open circuit (N_2_)	ND		ND		ND		ND		ND	

NM (1) = not measurable as YP is not a defined medium. NM (2) = not measurable as no net electron transfer occurs in the theoretical conversion of 3-hydroxybutyrate into crotonic acid

*ND* not detectable, *NH*_*4*_*Cl* with ammonium chloride, *N*_*2*_ nitrogen-fixing condition. () = standard deviation

*P* values are between growth with NH_4_Cl and N_2_ fixing conditions

^a^Values from biological replicates *n* = 2, otherwise *n* = 3

#### Photoautotrophic PHB production using a poised electrode as an electron donor

**Fig. 3:**
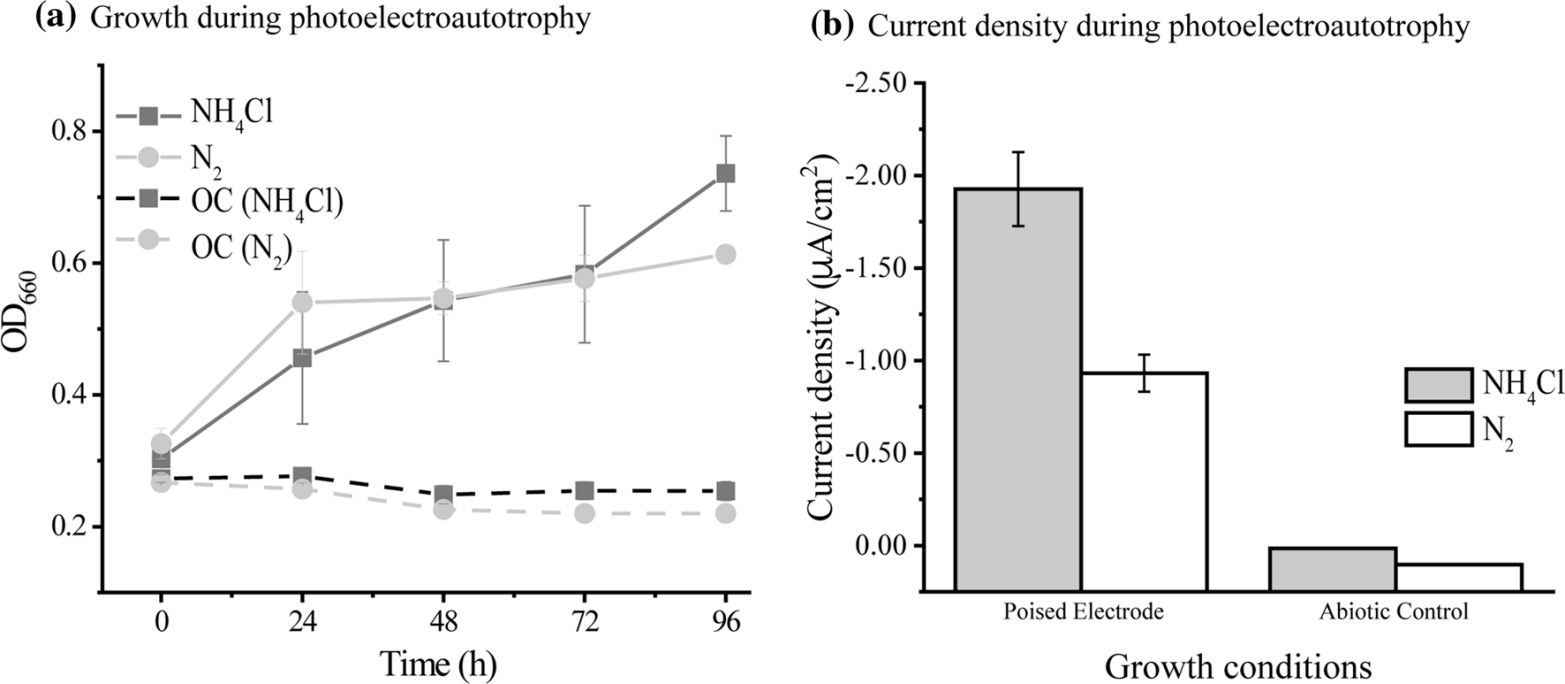
**a** TIE-1 growth during photoelectroautotrophy. OD_660_ values of TIE-1 grown under photoelectroautotrophy in freshwater medium (FW) with NH_4_Cl and under N_2_ fixing conditions with their respective control with unpoised electrodes (open circuit, OC). Error bars are from the standard deviations calculated using three biological replicates. **b** Current density during photoelectroautotrophy. Current density (µA/cm^2^) from TIE-1 grown with freshwater medium with NH_4_Cl as a nitrogen source or with N_2_ gas as a nitrogen source using a poised electrode at a potential of + 100 mV vs. Standard hydrogen electrode (SHE) and the associated abiotic control. The negative sign on the *Y*-axis indicates current uptake. Error bars are the standard deviations calculated using two biological replicates. The *P* values were determined by one-way ANOVA followed by a pairwise test with Bonferroni adjustment; *ns* not significant. For a pairwise test, the cutoff *P* value is 0.025. *P* values are indicated in Table S5

To evaluate PHB production under photoelectroautotrophy using poised electrodes as the sole electron donor, graphite electrodes were poised at a potential + 100 mV vs. SHE to mimic the Fe(OH)_3_/Fe^2+^ redox couple. Although the electrode–NH_4_Cl system resulted in higher maximum OD_660_ compared to the electrode–N_2_ system (0.73 vs. 0.61, respectively) (Table 1), there was no significant difference in the generation time of TIE-1 between these two conditions (Fig. 3a; Table 1). Nonetheless, after 96 h of incubation, EU by TIE-1 in the electrode–NH_4_Cl system was 1.92 µA/cm^2^, about double of that obtained in the electrode–N_2_ with an EU of 0.93 µA/cm^2^ (Fig. 3b, Table S6, *P* ≤ 0.01). Despite the difference in the total EU, no notable change was observed in the PHB carbon yield, PHB electron yield and specific PHB productivity between the electrode–NH_4_Cl system and the electrode–N_2_ system (Fig. 2a; Table 2). This result could be due to the continuous supply of electrons from a poised electrode, which did not seem to directly impact PHB biosynthesis. As expected, no cell growth was observed in the biotic reactors with unpoised electrodes (open circuit, OC) with both NH_4_Cl and N_2_ gas (Fig. 3a). Abiotic controls did not show any EU (Fig. 3b). Bioelectrosynthesis of PHB has been recently reported via a method using enzymatic and electrochemical approaches. A modified electrode poised at − 386 mV vs. SHE was used to synthesize NADH in the presence of enzymes of the PHB cycle to convert acetate to PHB. The amount of PHB produced was 0.3 mg/L (under a maximum current density (*J*_max_) of 27.9 ± 1.3 μA cm^−2^) [1]. In another study, 226 ± 6 mg/L of PHB was produced via indirect EU using formate as a mediator by *C. necator* (*Ralstonia eutropha*) at − 395 mV vs. SHE (~ *J*_max_ 213 μA cm^−2^) [12]. In addition, overexpression of the *ruBisCO* (Ribulose-1,5-bisphosphate carboxylase/oxygenase) gene was performed to increase the CO_2_ fixation [12]. As a result, PHB production was enhanced to 485 mg/L [12]. However, both of these approaches involve multiple steps including enzyme purification. They also operate at a higher reduction potential than what we use to grow TIE-1 for PHB production (+ 100 mV vs. SHE, *J*_max_ of only<2 μA cm^−2^). Although our results show a low (~ 5–6 mg/L) amount of PHB production under photoelectroautotrophy via direct MES, our approach offers numerous advantages: (1) using direct EU, we minimize the complexity of the MES system; (2) TIE-1 grows at a lower reduction potential than that used in the studies above. This lower reduction potential ultimately saves electrical energy; and (3) TIE-1 is a photoautotroph and, therefore, can use the energy of light to make excess ATP for biosynthesis. The photoautotrophic ability of TIE-1 makes is especially attractive for sustainable PHB bioproduction because light is an abundant resource. The major hurdle for using TIE-1 for bioproduction is the low electron uptake it demonstrates from graphite electrodes. This is reflected in the lower maximum current density (*J*_max_) values that were observed here in our study. Improving electron uptake would increase *J*_max_ values, which would also increase bioproduction [65]. A previous study from our laboratory has shown that inexpensive electrode modifications such as coating the electrodes with Prussian Blue can enhance electron uptake in the absence of a mediator via direct EU [65]. We are pursuing reactor design and electrode modifications further to enhance direct EU by TIE-1 because that will ultimately improve product formation.

#### Photoautotrophic PHB production using ferrous iron as an electron donor

**Fig. 4:**
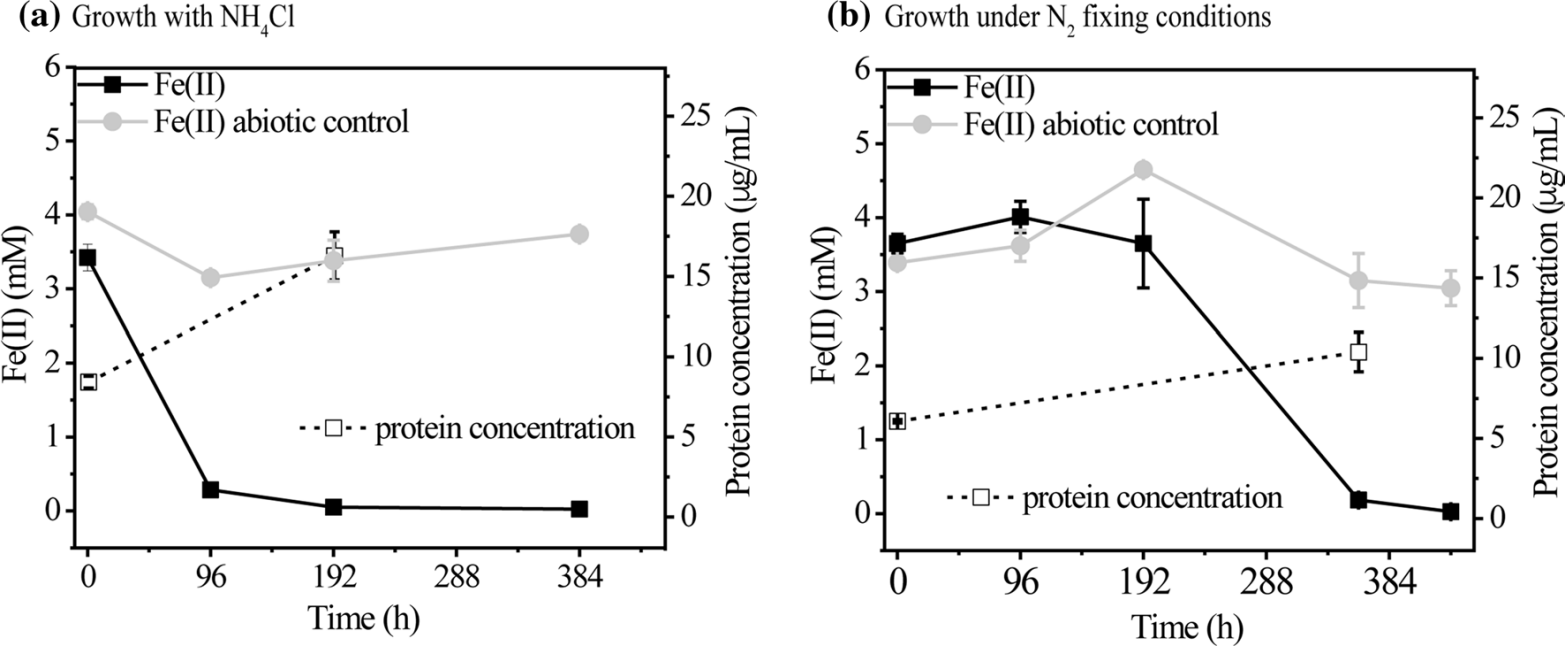
Growth of TIE-1 under photoferroautotrophic conditions. Fe(II) oxidation and protein concentration measured during photoferroautotrophy **a** with NH_4_Cl as a nitrogen source, and **b** with N_2_ gas as a nitrogen source. Error bars are from the standard deviations calculated using three biological replicates. The *P* values were determined by one-way ANOVA followed by a pairwise test with Bonferroni adjustment; *ns* not significant. For a pairwise test, the cutoff *P* value is 0.025). *P* values are indicated in Table S5

Specific PHB productivity was higher in the H_2_–N_2_ compared to the H_2_–NH_4_Cl systems (Table 2) but no difference was observed under photoelectroautotrophy. However, under photoferroautotrophy, the specific PHB productivity was higher in the Fe(II)–NH_4_Cl system compared to the Fe(II)–N_2_ (Table 2). In addition, PHB carbon yield decreased in the Fe(II)–N_2_ system compared to the Fe(II)–NH_4_Cl system (Fig. 2a; Table 2). By carefully examining Fe(II) oxidation in Fe(II)–NH_4_Cl system, a significant drop in Fe(II) concentration via microbial Fe(II) oxidation was observed during the first 96 h, and Fe(II) was completely oxidized by 384 h (Fig. 4a). In contrast, the Fe(II)–N_2_ system showed a very slow Fe(II) oxidation where a significant drop in Fe(II) concentration was observed only after 360 h (Fig. 4b). In addition, there was a decrease in the maximum OD_660_ in the Fe(II)–N_2_ system compared to the Fe(II)–NH_4_Cl system (Table 1). Additionally, total protein concentration with the Fe(II)–NH_4_Cl nearly doubled after 192 h whereas, with the Fe(II)–N_2_, the total protein concentration doubled only after 360 h (Fig. 4a, b). Based on these results, it is plausible that the growth defect in the Fe(II)–N_2_ system is a consequence of the high-energy demand during N_2_ fixation (Table 1; Fig. 4).

#### Comparison of PHB production under photoautotrophic conditions

Among the three electron donors tested, photoautotrophic growth with H_2_ showed the highest maximum OD_660_ when grown with NH_4_Cl (Table 1, *P* ≤ 0.001). Although the Fe(II)–NH_4_Cl and the Fe(II)–N_2_ conditions resulted in the lowest maximum OD_660_ (Table 1, *P* ≤ 0.001), the PHB carbon yield under the Fe(II)–NH_4_Cl is comparable to that obtained from photoautotrophy in the H_2_–NH_4_Cl system (Table 2, *P *=0.0613) and higher than under the electrode–NH_4_Cl (Table 2, *P *= 0.028). Moreover, the specific PHB productivity obtained in the Fe(II)–NH_4_Cl is the highest amongst all the photoautotrophic conditions with NH_4_Cl (Table 2, *P* ≤ 0.001). The electrode–N_2_ system showed the longest generation times amongst all the photoautotrophic growth conditions (Table 1, *P* ≤ 0.001). Photoelectroautotrophy showed the lowest PHB carbon yield both with NH_4_Cl and N_2_ (Fig. 2a, *P *= 0.0045). Nevertheless, TIE-1 showed the highest efficiency in converting electrons to PHB in the electrode–NH_4_Cl system (4.39% PHB electron yield) and in the electrode–N_2_ system (7.34% PHB electron yield) (Table 2, *P* ≤ 0.001). These results indicate that although the growth of TIE-1 during photoelectroautotrophy was slow, this condition was the most efficient at converting electrons obtained from a poised electrode into PHB. A previous comparative growth study of TIE-1 using H_2_ and soluble Fe(II) as electron donors revealed H_2_ as a preferred electron donor over Fe(II) [32]. This preference was reflected in the lower PHB carbon yield during photoferroautotrophy vs. photoautotrophy with H_2_ (Fig. 2; Table 2).

#### Effect of N_2_ fixation on photoautotrophic PHB production

The effect of N_2_ fixation was clearly observed in both H_2_ and Fe(II) systems, where cell growth and the PHB carbon yield was significantly reduced under N_2_ fixing conditions (Tables 1, 2). Based on previous studies, it is likely that the stress caused by the high-energy N_2_ fixation process led to the accumulation of PHB by TIE-1 [29, 51]. However, N_2_ fixation did not have a significant impact on the growth of TIE-1, the specific PHB productivity and the PHB carbon yield under photoelectroautotrophy (Table 2). A continuous electron supply might have allowed TIE-1 to fix N_2_ to ammonium without affecting the supply of electrons to produce PHBs. Interestingly, PHB biosynthesis was proposed to be a potential electron sink when *Rhodopseudomonas palustris* CGA009 was incubated in the presence of argon (nitrogen deprived) [51]. However, our results show that only a small percentage of electrons go to PHB biosynthesis under all N_2_ fixing conditions (Table 2). In the electrode–N_2_ system, where the maximum PHB electron yield was obtained, only 7.34% of the available electrons contributed to PHB biosynthesis.

### TIE-1 produces PHB under chemoheterotrophic and photoheterotrophic conditions

#### Chemoheterotrophic PHB production

To further investigate the effect of different media on PHB production, TIE-1 was grown chemoheterotrophically (aerobic) with rich media containing yeast extract and peptone (YP). Aerobic growth of TIE-1 on YP resulted in the longest generation time (*g* = 11.18 h) compared to the generation time observed from all the other photoheterotrophic conditions (Table 1, *P* ≤ 0.001). YP-grown cells produced the highest specific PHB productivity of 24.60 × 10^−14^ mg/L/Cell/h compared to all the conditions tested (Table 2, *P* ≤ 0.001). It is likely that the amino acids provided by peptone contributed to the increased PHB production by TIE-1 under this condition. The availability of amino acids precludes their de novo biosynthesis, and peptone has been previously reported to increase PHB production in *Azotobacter vinelandii* [56].

#### Photoheterotrophic PHB production with succinate

To test the effect of different carbon sources with different oxidation/reduction values (Supplemental Table S4) on PHB production, TIE-1 was further grown photoheterotrophically (anaerobic) using three different substrates: succinate, butyrate, and 3-hydroxybutyrate with NH_4_Cl and N_2_ gas. Similar to photoautotrophy, use of N_2_ gas as the source of nitrogen resulted in longer lag time, longer generation time and a longer time to reach maximum OD_660_ compared to NH_4_Cl as the nitrogen source (Table 1). However, there was no significant difference in specific PHB productivity between the succinate–N_2_ system and the succinate–NH_4_Cl system (Table 2). Rather there was an increase in the PHB carbon yield and PHB electron yield in the succinate–N_2_ system compared to the succinate–NH_4_Cl system (Fig. 2b; Table 2) indicating that there was no significant effect of N_2_ fixation on PHB productivity (Table 2).

#### Photoheterotrophic PHB production with butyrate and 3-hydroxybutyrate

Interestingly, TIE-1 grown photoheterotrophically with the less oxidized substrate, butyrate, showed a decrease in maximum OD_660_ (0.42) in the butyrate–NH_4_Cl compared to 0.69 with the butyrate–N_2_ systems (Table 1). An increase of about ninefold in PHB carbon yield was obtained in the butyrate–N_2_ system compared to the butyrate–NH_4_Cl system (Fig. 2b; Table 2). Similarly, specific PHB productivity in the butyrate–N_2_ system was more than three times higher compared to the butyrate–NH_4_Cl system (Table 2). The highest PHB production (~ 17.1 mg/L), as well as the highest PHB carbon yield, was obtained in the butyrate–NH_4_Cl system (Table 2). Although this PHB production is lower than the PHB production previously reported by another photosynthetic purple bacterium, *Rhodobacter sphaeroides* (60 mg/L) grown in olive mill wastewater under N_2_-limited conditions [22], TIE-1’s ability to produce PHB under various conditions such as photoautotrophy offers an obvious advantage when considering mixotrophic (photoheterotrophy and photoautotrophy) growth conditions for PHB production. Butyrate has been previously reported to be a preferred substrate over acetate in a PHB-producing mixed culture dominated by *Plasticicumulans acidivorans* due to the lower ATP need for PHB production using butyrate [49]. In addition, a study performed on *C. necator* has shown that butyrate is metabolized into 3-hydroxybutyryl-CoA via the beta-oxidation pathway. 3-Hydroxybutyryl-CoA is a direct precursor for PHB biosynthesis [16]. This shorter pathway might explain the higher PHB production along with faster generation time and a higher maximum OD_660_ obtained from butyrate compared to succinate and 3-hydroxybutyrate (Table 1; Table 2). Moreover, the higher numbers of electrons in butyrate compared to succinate and 3-hydroxybutyrate could contribute to higher PHB production in TIE-1 [29, 51]. A previous study by Shi et al. also reported similar results using metabolic flux balance analysis of PHB biosynthesis by *C. necator* under nitrogen-limited conditions using butyrate [71].

When TIE-1 was grown photoheterotrophically in 3-hydroxybutyrate, shorter lag time was observed in the 3-hydroxybutyrate–NH_4_Cl compared to the 3-hydroxybutyrate–N_2_ (Table 1). Although the maximum OD_660_ values were similar, N_2_ fixing conditions significantly increased the time to reach the maximum OD_660_ values (Table 1). A decrease in PHB electron yield was observed when TIE-1 was grown in the 3-hydroxybutyrate–NH_4_Cl system compared to the 3-hydroxybutyrate–N_2_ (Table 2). However, no significant differences were observed in the PHB carbon yield and specific productivity under these conditions (Table 2). These results indicate that N_2_ fixation slowed the growth of TIE-1 in 3-hydroxybutyrate but increased the PHB electron yield similar to the results obtained under other heterotrophic growth conditions (Table 1; Table 2).

#### Effect of N_2_ fixation on photoheterotrophic PHB production

Overall, N_2_ fixing conditions during photoheterotrophy delayed cell growth and resulted in a longer lag time as well as a longer time to achieve maximum OD_660_ (two times or longer) (Table 1). In contrast, PHB carbon yield under photoheterotrophy under N_2_ fixing conditions was higher than with NH_4_Cl (Table 2). This increase in PHB production under N_2_ fixing or N_2_ deprivation conditions is consistent with previous findings [29, 51]. This trend was not observed under photoautotrophic conditions. Interestingly, our results show that only a small percentage of carbon from the different substrates contributes to PHB synthesis by TIE-1. For example, the maximum PHB carbon yield during photoheteroautotrophic growth with butyrate was 8.81%. This indicates that the remaining carbon is likely used for biomass production. A previous report has shown that *R. palustris* grown on acetate converts 93% of its carbon into biomass [50].

### STEM–EELS confirms intracellular accumulation of PHB granules in TIE-1

**Fig. 5:**
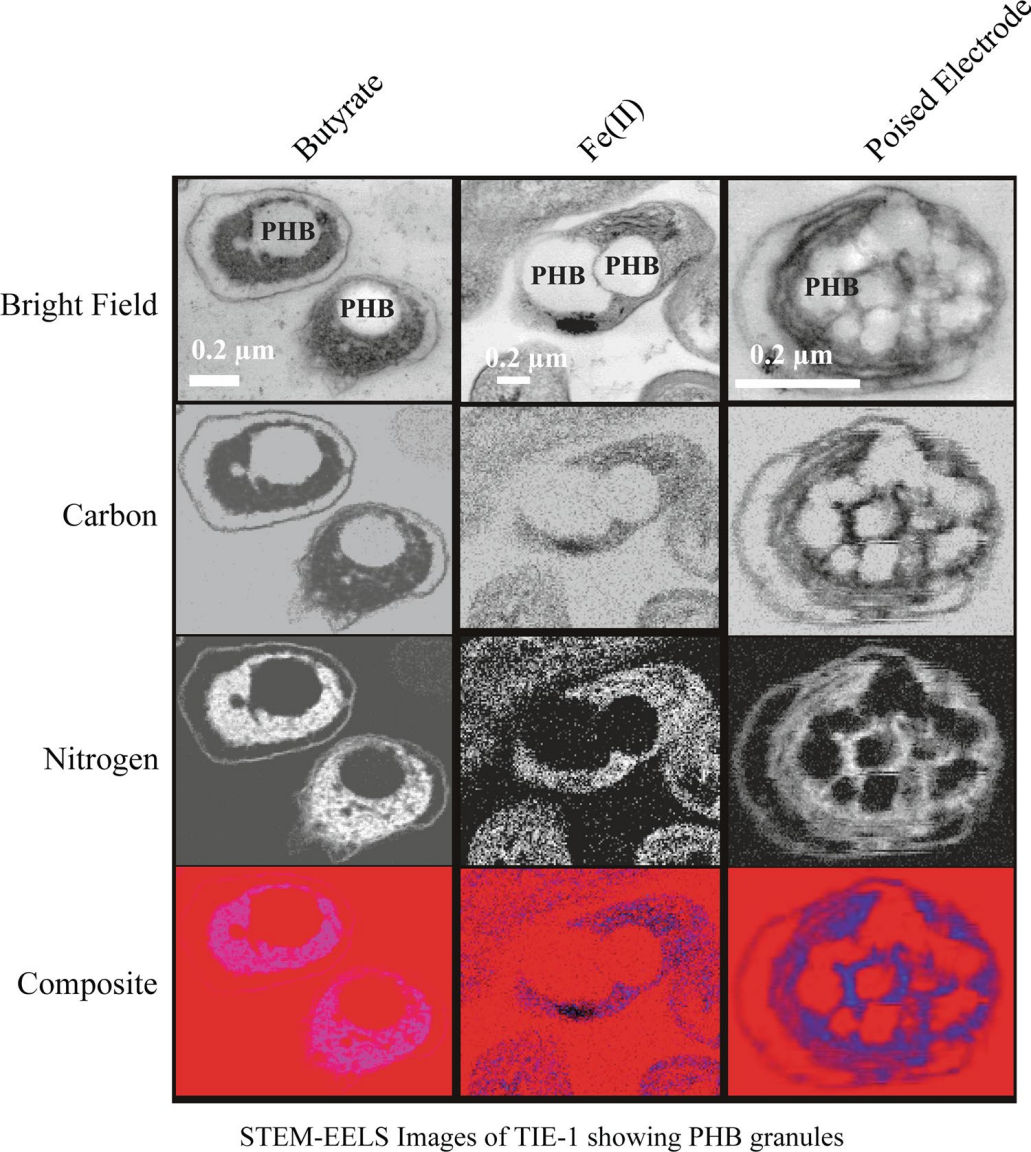
STEM–EELS images of TIE-1 grown photoheterotrophically with butyrate, photoferroautotrophically with Fe(II) and photoelectroautotrophically with a poised electrode. From top to bottom panel: bright-field image, carbon, and nitrogen map, and the composite images. Bright areas represent the dominance of the corresponding element (carbon, and nitrogen). The red background in the composite images is due to carbon signals from Spurr’s resin used for embedding the cells during sample preparation. The scale bars are 0.2 μm

Because the LC–MS method used for PHB quantification involves digestion of the PHB polymer into crotonic acid [35], it was necessary to confirm the presence of intracellular PHB granules in TIE-1 using an additional technique. Nile red staining has previously been used to screen for PHB and fatty acid esters in bacteria [61]. However, this staining technique was ineffective in showing any intracellular inclusion bodies in TIE-1 possibly due to its small size. Hence, scanning transmission electron microscopy-electron energy loss spectroscopy (STEM–EELS) under conditions that produced the highest accumulation of PHB (mg/L/Cell) was performed. All the conditions imaged contained NH_4_Cl. The intracellular localization of PHB granules was confirmed by the carbon and nitrogen maps (Fig. 5). The nitrogen signal is likely from the phasin protein that is known to bind PHB granules [63]. PHB mostly aggregated as small multiple granules under photoelectroautotrophy compared to larger granules under photoferroautotrophy and photoheterotrophy with butyrate (Fig. 5). A change in the number and morphology of PHB granules was also observed previously in an anoxygenic phototrophic bacteria *Dinoroseobacter sp.* JL1447 when it was grown with different carbon sources such as sodium acetate, glucose, sodium glutamate, sodium pyruvate, and trisodium citrate [85]. Moreover, in a study on a purple non-sulfur bacterium *Rhodovulum visakhapatnamense*, a change in size and an increase in the number of PHB granules were also observed under nitrogen stress [29].

### PHB cycle genes are not differentially expressed

**Fig. 6:**
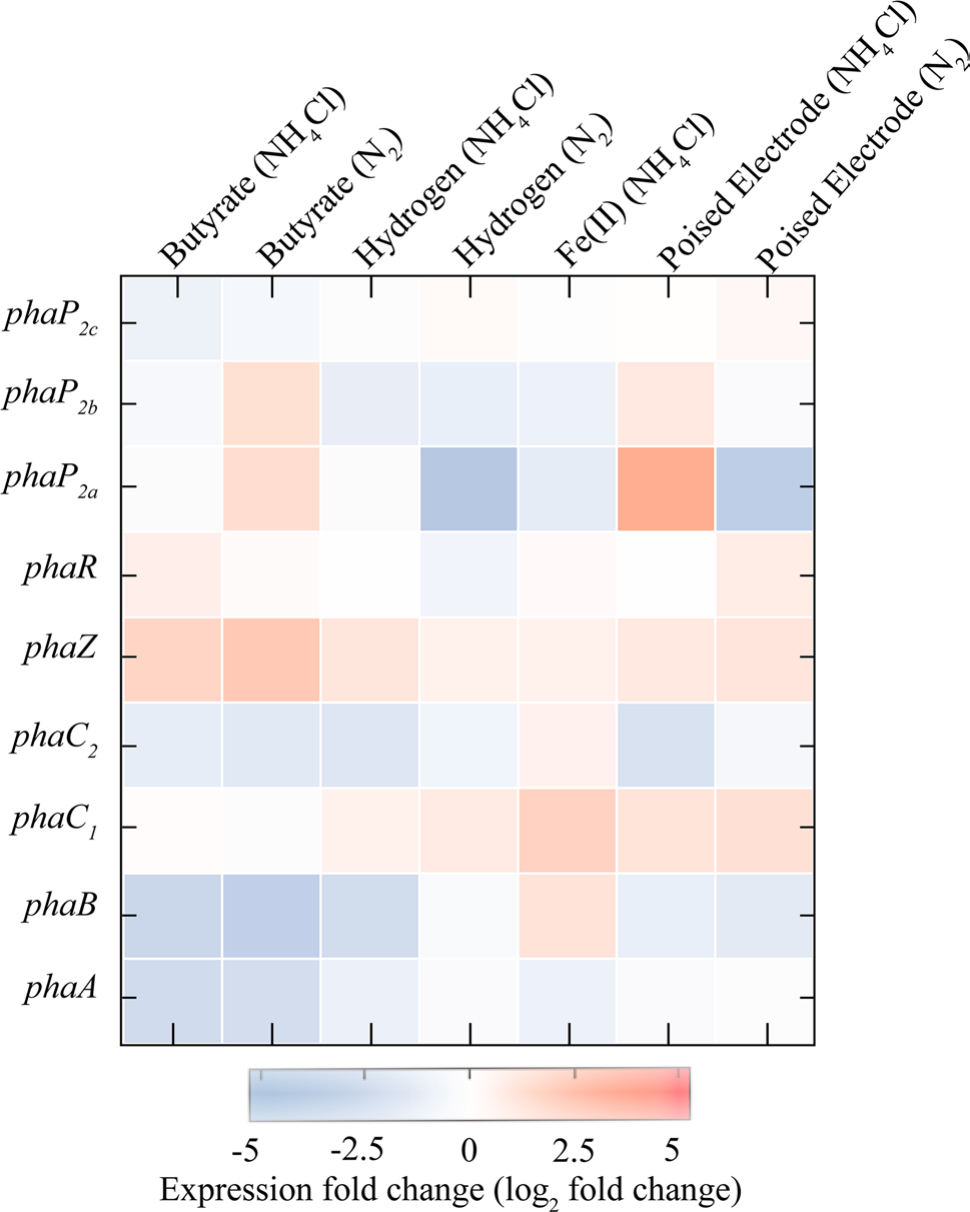
Heat map showing log_2_ fold change in the expression of PHB genes from RNA sequencing analysis (RNASeq). Results are from TIE-1 grown in freshwater (FW) medium photoheterotrophically with butyrate, photoautotrophically with H_2_, photoferroautotrophically with Fe(II), and photoelectroautotrophically using a poised electrode. Growth with NH_4_Cl is indicated by NH_4_Cl whereas growth under N_2_ fixing conditions is indicated by N_2_. Error bars are the standard deviations calculated using three biological replicates. The *P* values were determined by one-way ANOVA followed by a pairwise test with Bonferroni adjustment; *ns* not significant. For a pairwise test, the cutoff *P* value is 0.025). *P* values are indicated in Table S7–9

Our data show that there is significant variation in PHB production by TIE-1 under different growth conditions (Fig. 2; Table 2). To determine if genes involved in PHB production are transcriptionally regulated, transcript levels of the genes involved in the PHB cycle was assessed using RNA-Seq and reverse transcription quantitative PCR (RT-qPCR). PHB cycle genes were chosen and are summarized in Fig. 1a. RNA-Seq analysis showed that the PHB cycle genes were not differentially expressed with respect to growth conditions or levels of PHB (values having *p* > 0.05 are statistically not significant) (Fig. 6, Tables S7–S10). RT-QPCR was performed to corroborate these data. For this analysis, we chose one *phaA* isozyme (Rpal_0532) of many because it showed the highest expression in the RNAseq data. This gene exists in an operon with a *phaB* homolog (Rpal_0533) (Fig. 1b), which was the only *phaB* homolog that was chosen for RT-qPCR analysis. The other PHB cycle genes only had 1–2 representatives in the TIE-1 chromosome and these were all analyzed using RT-qPCR. RNAseq analysis combined with RT-qPCR analysis of these *phaAB* homologs (Table S11) revealed no significant changes in gene expression (Figure S1; Tables S8, S9). Moreover, the analysis of *phaC*_*1*_ and *phaC*_*2*_ polymerase genes did not show any significant differential expression (Fig. 6, Tables S7, S10). McKinlay et al. reported that *R. palustris* CG009 when incubated photoheterotrophically with acetate, under N_2_ deprivation (with argon), accumulated PHBs with no change in transcript levels of genes known to be involved in PHB biosynthesis [51].

Phasin proteins have been reported to play a significant role in the PHB cycle [86]. Deletion of the *phaP* gene reduced PHB production significantly in *C. necator* [86]. Here, no significant upregulation of *phaP*_*2*_ genes was observed using RNAseq even during photoheterotrophic growth with the butyrate–N_2_ system, where the highest PHB production in mg/L/cell was obtained (Supplemental Table S7). Although *phaP*_*2a*_ appears to show a slight upregulation of 2.33-fold change under photoelectroautotrophy with N_2_ fixing conditions, the *P* value was more than 0.05, rendering it not significant (Fig. 6, Table S7). Gene expression analysis results during photoautotrophy and photoheterotrophy using both RNAseq and RT-qPCR show that there is no differential expression in the genes involved in the PHB cycle. This could suggest that in TIE-1 (and perhaps even CGA009), the variation in PHB accumulation might be regulated post-transcriptionally. These findings are useful for the further optimization of PHB production using TIE-1 [45].

## Conclusions and future perspectives

Our study demonstrates the ability of a metabolically versatile photoautotroph *Rhodopseudomonas palustris* TIE-1 to produce PHB intracellularly under various growth conditions using different electron donors. The novel photoautotrophic metabolism using a poised electrode as the source of electrons produced the highest PHB electron yield. Another key discovery of this study is the ability of TIE-1 to yield the highest specific PHB productivity using Fe(II) as an electron donor for photoautotrophy. In summary, these newly described routes can serve as potential substitutes for PHB bioproduction. The application of these novel approaches can be especially important in areas where organic carbon sources are limited while resources such as light, CO_2_ [37], iron, and electricity [57] are abundant. TIE-1’s ability to fix N_2_ gas photoautotrophically makes it a more attractive biocatalyst for many applications including PHB biosynthesis. The extreme metabolic versatility of TIE-1 can also be considered for waste management efforts combined with MES (for example for PHB biosynthesis). This approach can be further scaled up using underwater tubular photobioreactors that have been used previously to investigate the photosynthetic efficiency of *R. palustris* 42OL [10]. The future of using biocatalysts like TIE-1 via direct EU for bioproduction needs further consideration. We are pursuing modifications of electrodes and changes in reactor design to improve direct EU by TIE-1 as this represents the first major hurdle in the application of such microbes for bioproduction [65]. Using TIE-1 in the context of photoferroautotrophy also needs further investigation as our data support the idea that biomolecule production can be linked to this process.

## Electronic supplementary material

Below is the link to the electronic supplementary material.

**Figure S1.** Heat map showing log_2_ fold change in the expression of PHB genes from reverse transcription quantitative PCR analysis (RT qPCR). Results are from TIE-1 grown in Fresh Water (FW) medium photoheterotrophically with butyrate, photoautotrophically with H_2_, photoferroautotrophically with Fe(II), and photoelectroautotrophically with a poised electrode. Growth with NH_4_Cl is indicated by NH_4_Cl whereas growth under N_2_ fixing conditions is indicated by N_2_. Error bars are the standard deviations calculated using 3 biological replicates. The *P* values were determined by one-way ANOVA followed by a pairwise test with Bonferroni adjustment; *ns: not significant*. For a pairwise test, the cutoff *P* value is 0.025). *P* values are indicated in Table S10. (TIFF 88863 kb)

Supplementary material 2 (DOCX 14 kb)

Supplementary material 3 (DOCX 12 kb)

Supplementary material 4 (DOCX 13 kb)

Supplementary material 5 (DOCX 12 kb)

Supplementary material 6 (DOCX 12 kb)

Supplementary material 7 (DOCX 23 kb)

Supplementary material 8 (DOCX 13 kb)

Supplementary material 9 (XLSX 11 kb)

Supplementary material 10 (XLSX 10 kb)

Supplementary material 11 (XLSX 11 kb)

Supplementary material 12 (XLSX 9 kb)

Supplementary material 13 (DOCX 16 kb)
