# Supplementary material for: Towards sustainable bioplastic production using the photoautotrophic bacterium Rhodopseudomonas palustris TIE-1
Source: J Ind Microbiol Biotechnol. 2019 Mar 29;46(9):1401–17. doi: 10.1007/s10295-019-02165-7 (PMC6791910; doi:10.1007/s10295-019-02165-7)
Supplement: Supplementary file 2 — Supplementary material 2 (DOCX 14 kb) [file 10295_2019_2165_MOESM2_ESM.docx]

**Supplemental methods**

**PHB extraction and analysis**

PHB extraction was performed as described previously with slight modification [35]. Briefly, samples were dried for 6 hours under vacuum in a Savant SC210A Speedvac concentrator (Thermo Fisher Scientific Inc, USA). 425 µL of methanol, 500 µL of HPLC grade chloroform and 75 µL of 95-98% sulfuric acid were added to the dried samples. Samples were digested for 1 hour in a water bath at 95°C. Digested samples were cooled on ice rapidly. 0.5 mL of LC-MS grade water was then added. Samples were vortexed and centrifuged at 5000 x *g* for 10 min. The organic phases were transferred into glass vials and dried for 30 min under speed vacuum. Finally, the dried samples were re-suspended in 500 µL of 50% acetonitrile + 50% water. Samples were filtered with 0.22 µm PTFE membrane filter to remove any cell debris prior to analysis. PHB measurements were performed using LC-MS.

Yield % ‘C’ mol PHB was calculated using the equation 1, 2 and 3 below:

$C mol substrate=\frac{Consumed substrate \left( \frac{g}{L} \right)\times Number of Carbon in substrate}{MW substrate}$(1)

$C mol PHB=\frac{PHB as crotonic acid \left( \frac{g}{L} \right)\times Number of Carbon in crotonic acid}{MW crotonic acid}$(2)

$Yield \% 'C' mol=100\times\frac{C mol PHB}{C mol substrate}$ (3)

The ‘Yield % ‘e^-^’ mol PHB’ was calculated from total electron available from each consumed electron donor as described below. Total available electron obtained from total oxidation of each organic acid to CO_2_ are shown in Supplemental Table S3. The inorganic electron donor Fe^2+^ and H_2_ release 1 and 2 electrons respectively as shown in Supplemental Table S3 adapted from [50]. Electrons supplied for electrotrophy growth were calculated directly from bioelectrochemical experiments wherein total current uptake was integrated over the operational time. Total electron uptake was used to calculate the Yield % ‘e^-^’ mol PHB because the electrode is the direct electron donor under this growth condition. This prevents us from using the oxidation/reduction method described below for Yield % ‘e^-^’ mol calculations. Electrons required for crotonic acid production was calculated from the oxidation/reduction value of the carbon in the conversion reaction of each substrate to crotonic acid. For example: in the conversion of succinate into crotonic acid, the oxidation/reduction value of succinate is +0.5, crotonic acid is -0.5, hence 4 mol e^-^ are involved; for butyrate, the oxidation/reduction value is -1, crotonic acid is -0.5, hence 2 mol e^-^ are involved; 3-hydroxybutyrate could not transfer any electron because of the its oxidation/reduction value similar to crotonic acid which is equal to -0.5. In the autotrophic growth conditions where CO_2_ is the carbon source, the oxidation/reduction value of CO_2_ is +4 which will involve 18 mol e^-^ in conversion to PHB. To obtain the number of electrons mol in the consumed substrate, the number of mol of consumed substrate was multiplied by the theoretical total available electron when the substrate is fully oxidized to CO_2_ (equation 4). To obtain the number of electrons required for PHB production, the number of mol of measured crotonic acid was multiplied by the theoretical number of electrons required for one mol of crotonic acid (equation 5). The obtained number was divided by the number of mol e^-^ consumed from each electron donor, multiplied by 100 (equation 6).

$e-mol substrate=Consummed substrate\left( mol \right)\times Total available electron in substrate$ (4)

$e-mol PHB=PHB as crotonic acid(mol)\times electron required for crotonic acid synthesis$ (5)

| $Yield \% 'e-'mol=100\times\frac{e-mol PHB}{e-mol substrate}$(6) |
| --- |

The theoretical values of available electron from various electron donors are listed in Supplemental Table S3.

Oxidation/Reduction values and theoretical electron required for crotonic acid synthesis are listed in Supplemental Table S4.
