# Supplementary material for: Towards sustainable bioplastic production using the photoautotrophic bacterium Rhodopseudomonas palustris TIE-1
Source: J Ind Microbiol Biotechnol. 2019 Mar 29;46(9):1401–17. doi: 10.1007/s10295-019-02165-7 (PMC6791910; doi:10.1007/s10295-019-02165-7)
Supplement: Supplementary file 3 — Supplementary material 3 (DOCX 12 kb) [file 10295_2019_2165_MOESM3_ESM.docx]

**Supplemental Table S1.** Primers used for checking DNA contamination.

| Primer descriptions | Locus | Gene Symbols | Primer sequences |
| --- | --- | --- | --- |
| phaC_1_ Forward cDNA | Rpal_2780 | *phaC1* | cttccagaacgaaatcatgcagctc |
| phaC_1_ Reverse cDNA | Rpal_2780 | *phaC1* | cgtcggaattccagtgcagc |
