# Supplementary material for: Towards sustainable bioplastic production using the photoautotrophic bacterium Rhodopseudomonas palustris TIE-1
Source: J Ind Microbiol Biotechnol. 2019 Mar 29;46(9):1401–17. doi: 10.1007/s10295-019-02165-7 (PMC6791910; doi:10.1007/s10295-019-02165-7)
Supplement: Supplementary file 4 — Supplementary material 4 (DOCX 13 kb) [file 10295_2019_2165_MOESM4_ESM.docx]

**Supplemental Table S2**. Primers used for reverse transcription quantitative PCR.

| Primer descriptions | Locus | Gene Symbols | Primer sequences |
| --- | --- | --- | --- |
| phaC_1_ Forward | Rpal_2780 | *phaC1* | gaacgcgagargaaggtacac |
| phaC_1_ Reverse | Rpal_2780 | *phaC1* | gacgtaggaccagatca |
| phaC_2_ Forward | Rpal_4722 | *phaC2* | gctgttcctggacacccgatc |
| phaC_2_ Reverse | Rpal_4722 | *phaC2* | atcttgaaggtcgatcgcca |
| phaR Forward | Rpal_0531 | *phaR* | cgaagatgatctgtgcgagc |
| phaR Reverse | Rpal_0531 | *phaR* | aacaccggcaccagtactta |
| phaA Forward | Rpal_0532 | *phaA* | tgtcggaagttttaacggc |
| phaA Reverse | Rpal_0532 | *phaA* | gaggatctggcccatgatca |
| phaB Forward | Rpal_0533 | *phaB* | attcggcagcggagaaattc |
| phaB Reverse | Rpal_0533 | *phaB* | gttgaccagaacatcgacgg |
| phaZ Forward | Rpal_0578 | *phaZ* | cccaacacctgcttcaactg |
| phaZ Reverse | Rpal_0578 | *phaZ* | atgctcgatcatctggtgga |
