# Supplementary material for: Towards sustainable bioplastic production using the photoautotrophic bacterium Rhodopseudomonas palustris TIE-1
Source: J Ind Microbiol Biotechnol. 2019 Mar 29;46(9):1401–17. doi: 10.1007/s10295-019-02165-7 (PMC6791910; doi:10.1007/s10295-019-02165-7)
Supplement: Supplementary file 5 — Supplementary material 5 (DOCX 12 kb) [file 10295_2019_2165_MOESM5_ESM.docx]

**Supplemental Table S3. Theoretical total available electron(s) mol/mol substrate**

| **Electron source** | **Total available electron(s)mol/Mol substrate** |
| --- | --- |
| Succinate | 14 |
| Butyrate | 20 |
| Hydroxybutyrate | 18 |
| Fe^2+^ | 1 |
| H_2_ | 2 |
