# Supplementary material for: Towards sustainable bioplastic production using the photoautotrophic bacterium Rhodopseudomonas palustris TIE-1
Source: J Ind Microbiol Biotechnol. 2019 Mar 29;46(9):1401–17. doi: 10.1007/s10295-019-02165-7 (PMC6791910; doi:10.1007/s10295-019-02165-7)
Supplement: Supplementary file 6 — Supplementary material 6 (DOCX 12 kb) [file 10295_2019_2165_MOESM6_ESM.docx]

**Supplemental Table S4. Oxidation/Reduction values and theoretical electrons required for crotonic acid synthesis**

| **Electron source** | **Oxidation/reduction value** | **Electrons required for crotonic acid synthesis** |
| --- | --- | --- |
| Succinate | +1 | 4 |
| Butyrate | -1 | 2 |
| Hydroxybutyrate | -0.5 | Not thermodynamically calculable |
| CO_2_ | +4 | 18 |
| Crotonic acid | -0.5 | Not applicable |
