# Supplementary material for: Towards sustainable bioplastic production using the photoautotrophic bacterium Rhodopseudomonas palustris TIE-1
Source: J Ind Microbiol Biotechnol. 2019 Mar 29;46(9):1401–17. doi: 10.1007/s10295-019-02165-7 (PMC6791910; doi:10.1007/s10295-019-02165-7)
Supplement: Supplementary file 7 — Supplementary material 7 (DOCX 23 kb) [file 10295_2019_2165_MOESM7_ESM.docx]

Supplemental Table S5. Statistical analysis of data from various growth condition using One-way ANOVA and pairwise t- test with Bonferroni adjustment

Supplemental Table S5a. *P* values of Max OD_660_ from different growth conditions comparison.

| Growth Conditions/Parameters/ *P* values | *P* values for Max OD_660_ |
| --- | --- |
| Hydroxybutyrate (N_2_) | 5.880 E-06 |
|  |  |
| Fe (II) (N_2_) |  |
| Photoelectroautotrophy (N_2_) |  |
| Hydroxybutyrate (NH_4_Cl) | 3.010 E-10 |
|  |  |
| Hydroxybutyrate (N_2_) |  |
| Fe (II) (NH_4_Cl) |  |
| Fe (II) (N_2_) |  |
| Photoelectroautotrophy (NH_4_Cl) |  |
| Photoelectroautotrophy (N_2_) |  |
| Hydrogen (NH_4_Cl) | 3.93E-06 |
| Fe (II) (NH_4_Cl) |  |
| Electrotrophy (NH_4_Cl) |  |
| Hydrogen N_2_ | 1.71E-05 |
| Fe (II) N_2_ |  |
| Electrotrophy N_2_ |  |
| Hydrogen (NH_4_Cl) | 3.58E-10 |
| Fe (II) (NH_4_Cl) |  |
| Electrotrophy (NH_4_Cl) |  |
| Hydrogen N_2_ |  |
| Fe (II) N_2_ |  |
| Electrotrophy N_2_ |  |

Supplemental Table S5b. *P* values of Yield % 'C' mol PHB from different growth conditions comparison.

| Growth Conditions/Parameters/ P values | *P* values for PHB carbon yield |
| --- | --- |
| Hydrogen (NH_4_Cl) | 0.0613 |
| Fe (II) (NH_4_Cl) |  |
| Hydrogen (NH_4_Cl) | 0.009 |
| Electrotrophy (NH_4_Cl) |  |
| Fe (II) (NH_4_Cl) | 0.0284 |
| Photoelectroautotrophy (NH_4_Cl) |  |
| Fe (II) (NH_4_Cl) | 0.008 |
| Fe (II) (N_2_) |  |
| Butyrate (N_2_) | 0.0697 |
| Hydrogen (NH_4_Cl) |  |
| Butyrate (N_2_) | 0.0173 |
| Fe (II) (NH_4_Cl) |  |
| Butyrate (N_2_) | 0.0023 |
| Photoelectroautotrophy (NH_4_Cl) |  |
| Succinate (N_2_) | 6.407E-05 |
| Butyrate (N_2_) |  |
| Butyrate (N_2_) | 0.000 |
| 3-Hydroxybutyrate (N_2_) |  |
| Succinate (N_2_) | 0.050 |
| 3-Hydroxybutyrate (N_2_) |  |
| Succinate (NH_4_Cl) | 8.280E-08 |
| Succinate (N_2_) |  |
| Butyrate (NH_4_Cl) |  |
| Butyrate (N_2_) |  |
| 3-Hydroxybutyrate (NH_4_Cl) |  |
| 3-Hydroxybutyrate (N_2_) |  |
| Hydrogen (NH_4_Cl) | 0.00452108 |
| Fe (II) (NH_4_Cl) |  |
| Electrotrophy (NH_4_Cl) |  |
| Hydrogen N_2_ |  |
| Fe (II) N_2_ |  |
| Electrotrophy N_2_ |  |
| Hydrogen (NH_4_Cl) | 0.00452 |
| Fe (II) (NH_4_Cl) |  |
| Electrotrophy (NH_4_Cl) |  |
| Hydrogen N_2_ |  |
| Fe (II) N_2_ |  |
| Electrotrophy N_2_ |  |
| Hydrogen (NH_4_Cl) | 0.00454 |
| Fe (II) (NH_4_Cl) |  |
| Electrotrophy (NH_4_Cl) |  |
| Hydrogen N_2_ | 0.4275 |
| Fe (II) N_2_ |  |
| Electrotrophy N_2_ |  |
| Hydrogen N_2_ | 0.5581 |
| Electrotrophy N_2_ |  |
| Fe (II) N_2_ | 0.2614 |
| Electrotrophy N_2_ |  |
| Succinate (NH_4_Cl) | 0.0374 |
| Butyrate (NH_4_Cl) |  |
| 3-Hydroxybutyrate (NH_4_Cl) |  |
| Succinate (N_2_) | 1.82E-06 |
| Butyrate (N_2_) |  |
| 3-Hydroxybutyrate (N_2_) |  |
| Succinate (NH4Cl) | 0.0670 |
| Butyrate (NH4Cl) |  |
| Succinate (NH4Cl) | 0.0399 |
| 3-Hydroxybutyrate (NH4Cl) |  |
| Butyrate (NH4Cl) | 0.1659 |
| 3-Hydroxybutyrate (NH4Cl) |  |

Supplemental Table S5c. *P* values of generation time from different growth conditions comparison.

| Growth Conditions/Parameters/ *P* values | *P* values for Generation time (hrs) |
| --- | --- |
| YP | 0.000 |
| Succinate (NH_4_Cl) |  |
| Butyrate (NH_4_Cl) |  |
| 3-Hydroxybutyrate (NH_4_Cl) |  |
| Hydrogen (NH_4_Cl) | 1.62E-06 |
| Fe (II) (NH_4_Cl) |  |
| Electrotrophy (NH_4_Cl) |  |
| Hydrogen N_2_ |  |
| Fe (II) N_2_ |  |
| Electrotrophy N_2_ |  |

Supplemental Table S5d. *P* values of PHB specific productivity (mg/L/Cell/h) from different growth conditions comparison.

| Growth Conditions/Parameters/ *P* values | *P* values for Specific productivity  (PHB mg/l/cell/h) |
| --- | --- |
| Hydrogen (NH_4_Cl) | 8.91E-05 |
| Photoelectroautotrophy (NH_4_Cl) |  |
| Fe (II) (NH_4_Cl) |  |
| Photoelectroautotrophy (NH_4_Cl) | 0.100 |
| Photoelectroautotrophy (N_2_) |  |
| Fe (II) (NH_4_Cl) |  |
| YP | 5.69E-10 |
| Succinate (NH_4_Cl) |  |
| Butyrate (NH_4_Cl) |  |
| 3-Hydroxybutyrate (NH_4_Cl) |  |
| Hydrogen (NH_4_Cl) |  |
| Photoelectroautotrophy (NH_4_Cl) |  |
| Fe (II) (NH_4_Cl) |  |
| Butyrate (NH_4_Cl) | 0.242 |
| YP |  |

Supplemental Table S5e. *P* values of electron uptake (µA cm-2) from different growth conditions comparison.

| Growth Conditions/Parameters/ *P* values | *P* values for Electron uptake (µA cm^-2^) |
| --- | --- |
| Photoelectroautotrophy (NH_4_Cl) | 0.008 |
| Photoelectroautotrophy (N_2_) |  |

Supplemental Table S5f. *P* values of PHB (mg/L/Cell) from different growth conditions comparison.

| Growth Conditions/Parameters/ *P* values | *P* values for PHB (mg/L/Cell) |
| --- | --- |
| Succinate (NH_4_Cl) | 1.94E-05 |
| Butyrate (NH_4_Cl) |  |
| 3-Hydroxybutyrate (NH_4_Cl) |  |
| YP |  |

Supplemental Table S5g. *P* values of Yield ‘e- ‘mol PHB from different growth conditions comparison.

| Growth Conditions/Parameters/ *P* values | *P* values for PHB electron yield |
| --- | --- |
| Hydrogen (NH_4_Cl) | 2.12E-08 |
| Hydrogen (N_2_) |  |
| Electrotrophy (NH_4_Cl) |  |
| Electrotrophy (N_2_) |  |
| Fe (II) (NH_4_Cl) |  |
| Fe (II) (N2) |  |

Supplemental Table S5h. Anova statistical test on each parameter from all the growth condition: YP, Photoautotrophy using hydrogen, ferrous iron and electrode poised at sufficiently negative potential, photoheterotrophy using succinate, butyrate and 3- hydroxybutyrate with NH_4_Cl and under N_2_ fixing conditions.

| Parameters | Lag time (h) | Generation time(h) | Max OD660 | Time to achieve max OD_660_ (h) |
| --- | --- | --- | --- | --- |
| *P* values | 2.668E-16 | 4.603E-15 | 2.213E-15 | 6.425E-40 |
