# Supplementary material for: Towards sustainable bioplastic production using the photoautotrophic bacterium Rhodopseudomonas palustris TIE-1
Source: J Ind Microbiol Biotechnol. 2019 Mar 29;46(9):1401–17. doi: 10.1007/s10295-019-02165-7 (PMC6791910; doi:10.1007/s10295-019-02165-7)
Supplement: Supplementary file 8 — Supplementary material 8 (DOCX 13 kb) [file 10295_2019_2165_MOESM8_ESM.docx]

**Supplemental Table S6**. Mean maximum current density (*J*_max_), Total quantity of charge (C) and electron transferred (mol e^-^) during the growth of TIE-1 under photoelectroautotroph condition (*n*=2 biological replicates) in the presence of ammonium chloride or under nitrogen fixing condition (N_2_).

| System | Time (h) | Mean maximum Current density (*J_max_*, µA/cm^2^) | Total Coulombs transferred (C) | Number of electrons transferred (mol e^-^) |
| --- | --- | --- | --- | --- |
| Abiotic Control N_2_ | 96 | 0.103 (0.001) | -0.026 (0.003) | 2.7046 x 10^-7^ |
| *R. palustris* TIE-1 N_2_ | 96 | -0.932 (0.100) | -0.154 (0.017) | 15.979 x 10^-7^ |
| Abiotic Control + NH_4_Cl | 96 | 0.015 (0.002) | -0.012 (0.002) | 1.2331 x 10^-7^ |
| *R. palustris* TIE-1 + NH_4_Cl | 96 | -1.927 (0.2) | -20.454 (2.122) | 2119.6 x 10^-7^ |

()= standard deviation from *n*=2
