# Supplementary material for: Towards sustainable bioplastic production using the photoautotrophic bacterium Rhodopseudomonas palustris TIE-1
Source: J Ind Microbiol Biotechnol. 2019 Mar 29;46(9):1401–17. doi: 10.1007/s10295-019-02165-7 (PMC6791910; doi:10.1007/s10295-019-02165-7)
Supplement: Supplementary file 13 — Supplementary material 13 (DOCX 16 kb) [file 10295_2019_2165_MOESM13_ESM.docx]

Supplemental Table S11. Homolog of PhaA and PhaB

Homologs of PhaA (From Blast search)

| Gene annotation | Sequencing producing significant alignments | Max Score | Total Score | Query cover (%) | E value | Identity  (%) |
| --- | --- | --- | --- | --- | --- | --- |
| Rpal_0532 | acetyl-CoA C-acetyltransferase [*R. palustris*] | 792 | 792 | 100 | 0.0 | 100 |
| Rpal_0886 | acetyl-CoA C-acetyltransferase [*R. palustris*] | 817 | 817 | 100 | 0.0 | 100 |
| Rpal_1903 | acetyl-CoA C-acetyltransferase [*R. palustris*] | 800 | 800 | 100 | 0.0 | 100 |
| Rpal_1956 | acetyl-CoA C-acetyltransferase [*R. palustris*] | 1021 | 1021 | 100 | 0.0 | 100 |
| Rpal_2547 | acetyl-CoA C-acetyltransferase [*R. palustris*] | 797 | 797 | 100 | 0.0 | 100 |
| Rpal_3607 | thiolase domain-containing protein [*R. palustris*] | 782 | 782 | 100 | 0.0 | 100 |
| Rpal_4830 | acetyl-CoA C-acetyltransferase [*R. palustris*] | 839 | 839 | 100 | 0.0 | 100 |

Homologs of PhaB (From Blast search)

| Gene annotation | Sequencing producing significant alignments | Max Score | Total Score | Query cover (%) | E value | Identity  (%) |
| --- | --- | --- | --- | --- | --- | --- |
| Rpal_0113 | SDR family Oxidoreductase [*R.* *palustris*] | 522 | 522 | 100 | 0.0 | 100 |
| Rpal_0533 | beta-ketoacyl-ACP reductase [*R. palustris*] | 490 | 490 | 100 | 5e-175 | 100 |
| Rpal_0964 | SDR family Oxidoreductase [*R*. *palustris*] | 499 | 499 | 100 | 1e-178 | 100 |
| Rpal_1300 | Short-chain dehydrogenase/reductase SDR [*R. palustris*] | 479 | 4850 | 100 | 6e-152 | 92 |
| Rpal_1880 | SDR family Oxidoreductase [*R.* *palustris*] | 406 | 406 | 99 | 2e-157 | 87 |
| Rpal_3484 | 3-oxoacyl-[acyl-carrier-protein] reductase [*R. palustris*] | 492 | 492 | 100 | 8e-176 | 100 |
| Rpal_3838 | 3-oxoacyl-ACP reductase FabG [*R. palustris*] | 479 | 479 | 100 | 7e-171 | 100 |
| Rpal_5077 | SDR family NAD(P)-dependent oxidoreductase [*R. palustris*] | 474 | 474 | 100 | 7e-169 | 100 |
| Rpal_5138 | 3-oxoacyl-ACP reductase FabG [Rhodopseudomonas palustris] | 508 | 508 | 100 | 0.0 | 100 |
